# Supplementary material for: Phenolic-Compound-Rich Opuntia littoralis Ethyl Acetate Extract Relaxes Arthritic Symptoms in Collagen-Induced Mice Model via Bone Morphogenic Markers
Source: Nutrients. 2022 Dec 17;14(24):5366. doi: 10.3390/nu14245366 (PMC9783915; doi:10.3390/nu14245366)
Supplement: Supplementary file 1 [file nutrients-14-05366-s001.zip › nutrients-2043684-supplementary.pdf]

Table S1. Phytochemical analysis of the different extracts from Opuntia Littoralis cladodes.

Plant extracts	Hexane extract	EAE	HAE
**Carbohydrates and/or glycosides**	-	+	+
**Alkaloids**	-	+	+
**Tannins**	-	-	+
**Flavonoids**	±	+	+

(+) = Positive, (±) = Traces and (-) = Negative

Table S2. Phytochemical analysis of the different extracts from Opuntia Littoralis cladodes.

Plant extracts	Total phenolic (mg Gallic acid/gram)	Total flavonoid (mg Rutin /gram)
**Hexane Extract**	55.2 ± 2.1 ^{c}	19.4 ± 1.7 ^{c}
**EAE**	69.7 ± 3.7 ^{b}	53.2 ± 4.3 ^{a}
**70% HAE**	88.3 ± 7.1^{a}	45.2 ± 3.98 ^{ab}

Each value in the table is represented as mean ± SD (n = 3). Values in the same column followed by a different letter ($^{a-f}$) are significantly different ($p< 0.05$). -, not determined.

A

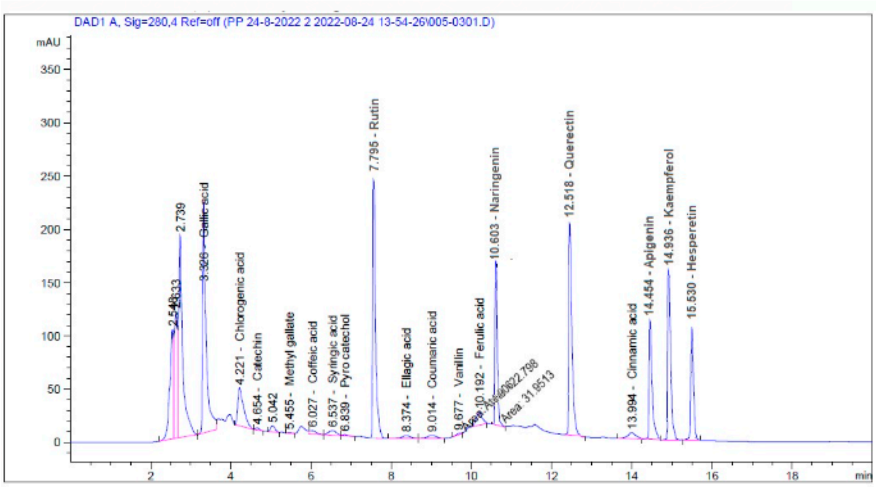

B

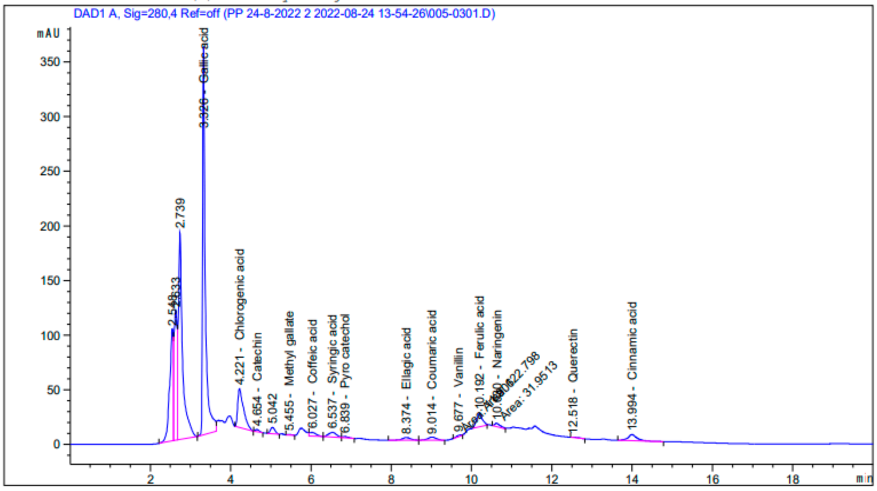

Figure S1. HPLC pattern analysis of the O. littorallis cladodes extracts: A) Ethyl acetate extract (EAE). B) Hydro alcoholic extract (HAE).

Table S3. Polyphenolic compounds concentration in Ethyl acetate and hydro alcoholic extracts of O. littorallis cladodes using HPLC.

Retention time (min)	Polyphenolic compounds	Concentration (µg/g)		Compound Nature
		EAE	HAE	
3.326	Gallic acid	2912.82	4874.53	Phenolic acid
4.221	Chlorogenic acid	1853.32	1756.29	
4.654	Catechin	86.85	111.42	Flavonol
5.455	Methyl gallate	4.26	6.77	Phenolic acid
6.027	Caffeic acid	71.65	105.93	
6.537	Syringic acid	96.11	187.45	
6.839	Pyro catechol	54.32	62.47	Phenolic compound
7.795	Rutin	3764.54	0.00	Flavonoid glycoside
8.374	Ellagic acid	102.26	304.84	Phenolic acid
9.014	Coumaric acid	40.15	41.37	phenolic derivative of cinnamic acid
9.677	Vanillin	6.14	13.81	phenolic aldehyde
10.192	Ferulic acid	46.27	242.09	Phenolic acid
10.630	Naringenin	2016.78	89.81	Flavanone
12.216	Daidzein	0.00	0.00	Hydroxyisoflavone
12.518	Quercetin	2469.22	18.71	Flavonol
13.994	Cinnamic acid	64.12	71.54	Phenolic acid
14.454	Apigenin	1223.34	0.00	Flavone
14.936	Kaempferol	1876.27	0.00	Flavonol
15.530	Hesperetin	984.68	0.00	Flavanone

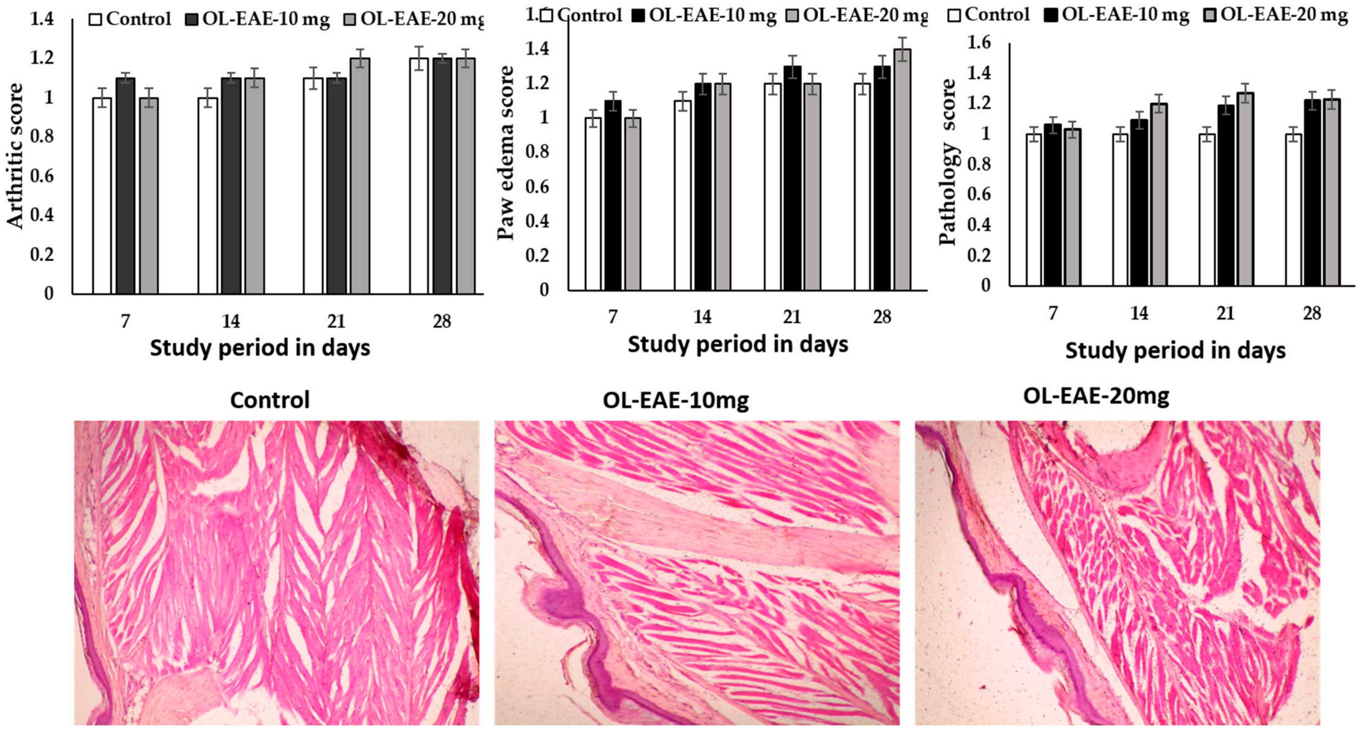

Figure S2: Arthritic score and pathology score OL-EAE treated naïve mice.

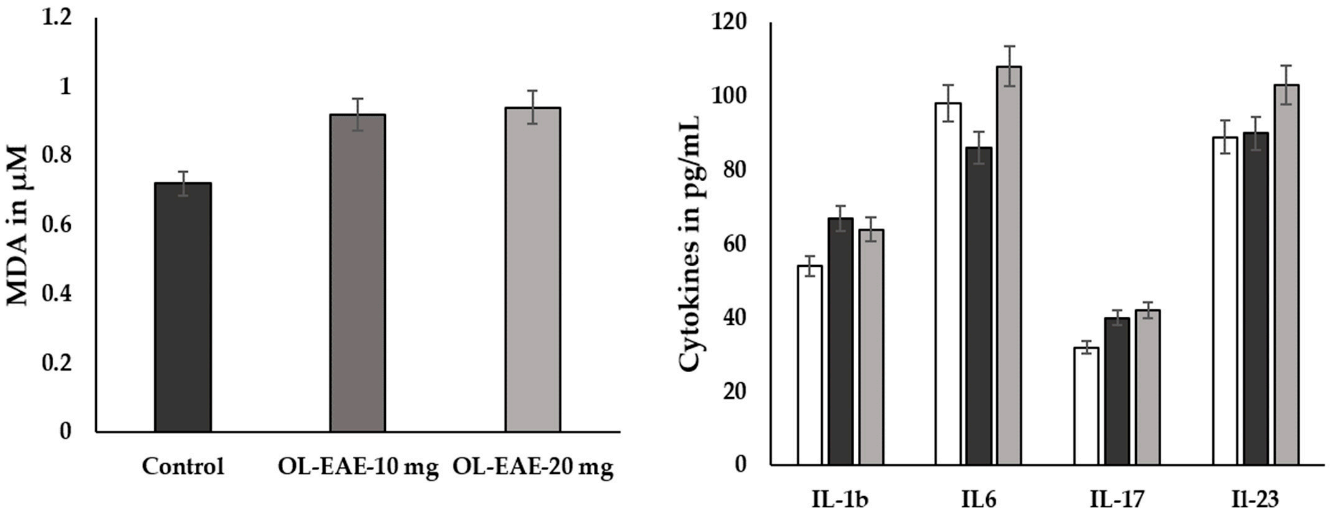

Figure S3: Oxidative stress marker and inflammatory cytokine levels in OL-EAE treated naïve mice
